# The Effect of Zr and Y on the Corrosion Behavior of T6-Treated Al–Si–Mg Alloys

**DOI:** 10.3390/ma18122705

**Published:** 2025-06-09

**Authors:** Pengcheng Ye, Feifei Wu, Feng Jiang

**Affiliations:** School of Materials Science and Engineering, Central South University, Changsha 410083, China; 193101010@csu.edu.cn (P.Y.); wff0523@163.com (F.W.)

**Keywords:** A356, rare earth, corrosion behavior, electrochemistry

## Abstract

The microstructure evolution and corrosion behavior of T6-treated Al–Si–Mg alloys were investigated in the presence of Zr and Y additions by using X-ray diffractometry (XRD), optical microscopy (OM), scanning electron microscopy (SEM), electrochemical measurement, and X-ray photoelectron spectroscopy (XPS). The results show that the coarse dendritic α-Al was refined into finer, equiaxed grains by adding 0.2 wt% Zr, which further promoted the formation of a uniform and dense passive layer in 3.5% NaCl solution at room temperature (≈25 °C). In contrast, the Al–Si–Mg alloy containing 0.3 wt% Y exhibited the highest corrosion rate. This phenomenon arises from converting rod-like eutectic Si particles into a spherical morphology, which exacerbates the intergranular corrosion network and enhances galvanic coupling effects.

## 1. Introduction

The Al–Si alloy is well-known for its commendable corrosion resistance and mechanical properties, which make it extensively used in the aerospace and automotive industries [1,2]. Introducing Mg into Al–Si binary alloys notably enhances their strength and hardness. This is attributed to the precipitation of Mg_2_Si, which functions as an effective strengthening phase [3]. The hypoeutectic Al–Si–Mg alloy exhibits excellent mechanical properties and thermal stability. However, untreated Al–Si–Mg casting alloys typically exhibit a large dendritic α-Al phase and coarse plate–like eutectic Si morphology. Research has shown that a passive layer naturally develops on the surface of Al–Si alloys, effectively preventing environmental corrosion [4]. However, the dendritic α-Al and coarse plate-like eutectic Si in unmodified Al–Si–Mg alloys can cause an uneven distribution of the passive film, leading to notable localized corrosion of the Al–Si–Mg alloy [5]. This corrosion problem significantly hinders the wider industrial use of Al–Si–Mg alloys, highlighting the need for material optimization.

Studies indicate that grain refinement’s impact on alloy corrosion resistance significantly depends on the environmental context, proving either beneficial or detrimental [6]. In active settings, smaller grain sizes generally decrease corrosion resistance, while in passive environments, they can enhance it. This is attributed to the promotion of protective passive film formation on exposed surfaces. For example, aluminum alloys tend to become passive in standard service environments like a 3.5% NaCl solution [7]. Thus, the refined α-Al dendrites can improve the corrosion resistance. Al–5Ti–B and Al–Ti–C are commonly used as grain refiners and added before casting, which can greatly refine the primary α-Al phases [8].

Moreover, the corrosion problems can be alleviated through the modification of eutectic silicon. By adding sodium (Na) and strontium (Sr) [9], as well as rare earth (RE) elements [10,11,12], the eutectic Si can be refined from a coarse plate-like structure into fine feathers. However, optimal effects in grain refinement and modification are only realized when both grain refiners and modifiers are added together. These effects often weaken after the alloy melts have been held for a long duration [13]. Moreover, when the concentrations of both Sr and B surpass a certain threshold upon simultaneous addition, a mutual poisoning effect takes place [14]. Incorporating 0.3 wt% Y into Al–7.5Si–0.5Mg alloys transforms the eutectic Si morphology from coarse plate-like to finer fibrous structures [15]. Liu et al. [16] demonstrated that incorporating Y into semi-solid A356 Al alloys refines the α-Al grain size and modifies the primary α-Al particles. Adding 0.3 wt% Er improves the corrosion resistance of the A356 alloy. This enhancement is due to the reduced volume fraction of intermetallic compounds in the eutectic region, leading to a decreased contact surface between the dendrites and the cathode region [17]. The ADC12 alloy was doped with 0.9 wt% Yb to form Al_3_Yb precipitates on the cathodic Si particles, thereby inhibiting their growth and effectively reducing the microgalvanic corrosion activity [18]. In Al−11Si−2Cu−0.8Zr die-cast alloys, the addition of Bi, Sb, and Sr effectively refines the eutectic Si. Nonetheless, this could negatively impact the alloy’s corrosion resistance [19]. The grain structure of Al–5.5Mg–0.3Mn alloys can be refined with the addition of 0.1 wt% Y and 0.1 wt% Zr, and the segregation of Mg during the casting process is also improved. In addition, the corrosion susceptibility can be reduced due to the formation of an AlMgYZr phase, which decreases the potential difference with the substrate [20]. According to Yu-kun MA et al. [21], adding Sc to Al−6.5Si−0.45Mg alloys improves their corrosion resistance in NaCl solutions more effectively than adding Sr, but excess Sc does not lead to additional benefits. On the other hand, the corrosion resistance of Al–6.5Si–0.45Mg was reduced due to the addition of Sc and Zr, which modified the eutectic Si particles and formed more galvanic couplings [22].

In Al–Si–Mg alloys, refining the grains and modifying the eutectic silicon are critical concerns. Given the demanding working conditions of these alloys, much research has been dedicated to enhancing their structure and mechanical properties. This focus on structural and mechanical improvements is also evident in studies involving Zr/Y additions. To address this situation, the impact of adding Zr/Y on the corrosion performance of the widely used A356 alloy in cast aluminum alloys after T6 treatment, as well as the potential corrosion mechanism, was investigated. This research could provide valuable insights for the subsequent design of Al–Si–Mg alloy compositions.

## 2. Experimental Procedure

Four groups were established: Al–7Si–0.3Mg (A0), Al–7Si–0.3Mg–0.2Zr (AZ2), Al–7Si–0.3Mg–0.3Y (AY3), and Al–7Si–0.3Mg–0.2Zr–0.3Y (AZ2Y3). The commercial A356 (produced by Zhejiang Extreme Aluminum New Material Co., Ltd., Taizhou, China), Al–4%Zr, and Al–10%Y master alloys were used in this study; the specific preparation steps can be found in [23]. The chemical composition was analyzed using ICP-AES, as shown in Table 1. The T6 heat treatment involves 535 °C for 5 h and aging at 180 °C for 5 h. Unless otherwise specified, the alloys in this text are assumed to be in the T6 state.

An X-ray diffractometer (XRD, Rigaku D/MAX-2550VB, Tokyo, Japan) was used to analyze the phase composition from 20° to 80° with a speed of 5°/min. Microstructural analysis was performed utilizing a Leica DFC295 optical microscope (OM, Wetzlar, Germany) to capture detailed surface images. Additionally, a JSM-7900F scanning electron microscope (SEM, JEOL, Tokyo, Japan) equipped with energy-dispersive spectrometry (EDS) was used to identify the elemental distribution of the samples.

Electrochemical characterization was conducted with a Multi-Autolab M204 workstation (Metrohm, LB Barendrecht, Netherlands), including open-circuit potential (OCP), electrochemical impedance spectroscopy (EIS) and potentiodynamic polarization curves. Measurement experiments used a three-electrode system. Samples with an area of 1 cm^2^ and total size of 1 cm × 1 cm × 0.5 cm were used as the working electrode. A platinum plate (10 mm × 10 mm × 0.5 mm) was used as the counter electrode and a saturated calomel electrode (SCE) as the reference electrode. The samples used for electrochemical experiments underwent mechanical grinding with SiC paper and polishing with diamond paste. The alloy samples were initially submerged in a 3.5% NaCl solution for 7 days, followed by immersion in an electrolyte for 900 s to monitor the OCP until stabilization. The potentiodynamic polarization curves were measured at a 2 mV/s scan rate over a potential range of −0.4 V to −1.1 V. The corrosion behavior of the alloy specimens was evaluated using the Tafel extrapolation method, with potential ranges for data fitting set between 60 and 120 mV. The Stern–Geary equation [24] allows for the calculation of corrosion current density (J_corr_) from polarization resistance (R_p_).

(1) $$J_{corr}=\frac{\beta_{a}\beta_{c}}{2.303R_{p}\left(\beta_{a}+\beta_{c}\right)}=\frac{B}{R_{p}}$$

β_a_ and β_c_ represent the anodic and cathodic Tafel slopes (calculated as modular value), respectively. The instantaneous corrosion rate P_i_ (mm/year) was derived from the J_corr_ value (A/cm^2^) based on the polarization curve [25].

(2) $$p_{i}=22.85J_{corr}$$

EIS measurements were carried out within the range of 0.01 Hz to 100 KHz, with an amplitude of 5 mV. The impedance spectra were analyzed using ZView3.1 software. Each sample underwent triplicate electrochemical corrosion tests to ensure experimental reliability.

An immersion corrosion experiment was conducted for 28 days in a 3.5% NaCl solution at room temperature of 25 °C. The sample size used in this experiment was 10 mm × 10 mm × 3 mm. The samples were removed from the solution every 7 days, and the corrosion products were removed using a solution comprising 20 g/L chromic oxide and 50 mL/L phosphoric acid at a temperature of (80 ± 2) °C for 3 min. The surface morphologies and elemental distribution of the alloy samples were examined using SEM after the immersion corrosion experiment.

X-ray photoelectron spectroscopy (XPS, Thermo Fisher Scientific K-ALPHA, Horsham, UK) was used to detect the chemical composition of the corrosion products. The X-ray spot size was 400 μm. The operating voltage was set to 15 kV with a filament current of 6 mA. The passing energy was set to 50 eV, with a step size of 0.1 eV, using a charge neutralizer for charge correction. The C 1s peak at 284.8 eV was employed as a reference to correct for charge offset, and the Avantage software (version 6.9) was utilized to fit the XPS experimental data.

## 3. Results and Discussion

### 3.1. Microstructure Studies

**Figure 1:**
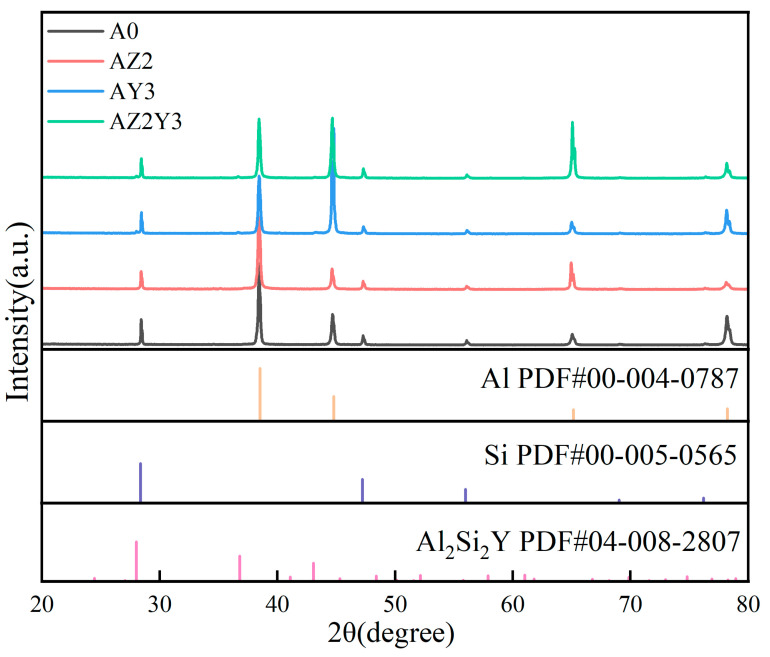
XRD patterns of four alloys.

Figure 1 shows the XRD patterns of four alloys. It can be seen that the main phases in the four alloys are α-Al and Si. The lack of Zr-rich peaks is due to the low Zr content and partial dissolution of Zr into the matrix during solution treatment. The introduction of Y led to the formation of an Al_2_Si_2_Y phase in both AY3 and AZ2Y3 alloys.

**Figure 2:**
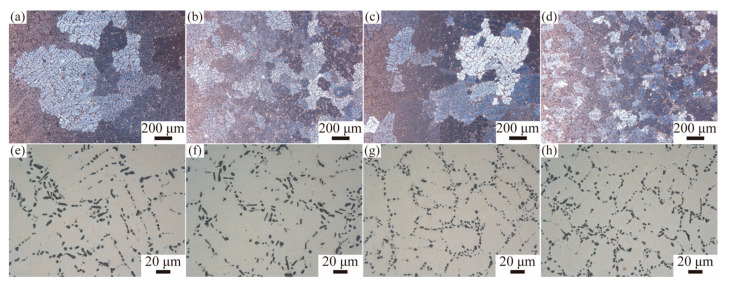
OM micrographs of four alloys: (**a**,**e**) A0, (**b**,**f**) AZ2, (**c**,**g**) AY3, and (**d**,**h**) AZ2Y3.

Figure 2 shows the optical microstructure of four T6-treated alloys. The inclusion of Zr and/or Y modified the size of the α-Al primary dendrites and eutectic Si particles (Figure 2a–d). In the A0 alloy (Figure 2a,c), the α-Al grains exhibit coarse dendritic structures, while the eutectic Si appears partially spherical and rod-shaped. Incorporating 0.2 wt% Zr into the A356 alloy significantly refines the α-Al grains, transforming them from coarse dendrites into smaller spherical forms (Figure 2b,d). This alteration is due to the heterogeneous nucleation of pro-peritectic (Al, Si)3(Zr, Ti). The eutectic Si in the AZ2 alloy exhibits minimal variation compared to the A0 alloy. The addition of 0.3 wt% Y primarily converted eutectic Si particles into fine spherical shapes due to Y-containing intermetallics forming at the grain boundaries (see Figure 2c,e). The simultaneous addition of Zr and Y leads to the refinement of α-Al grains and the modification of eutectic Si, as documented in the literature [25,26]. An evaluation of the effectiveness of Si modification due to Y and/or Zr additions is summarized in Table 2. The aspect ratios for the alloys are as follows: A0 alloy is 2.45 ± 1.61, AZ2 is 2.46 ± 1.48, AY3 is 1.41 ± 0.36, and AZ2Y3 is 1.52 ± 0.49.

**Figure 3:**
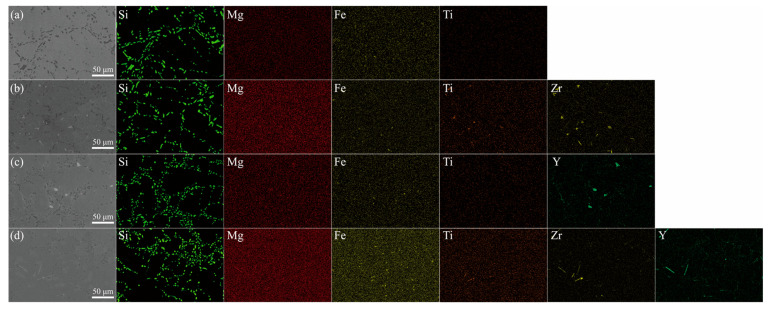
SEM micrographs of four alloy samples: (**a**) A0 alloy, (**b**) AZ2 alloy, (**c**) AY3 alloy, and (**d**) AZ2Y3 alloy.

**Figure 4:**
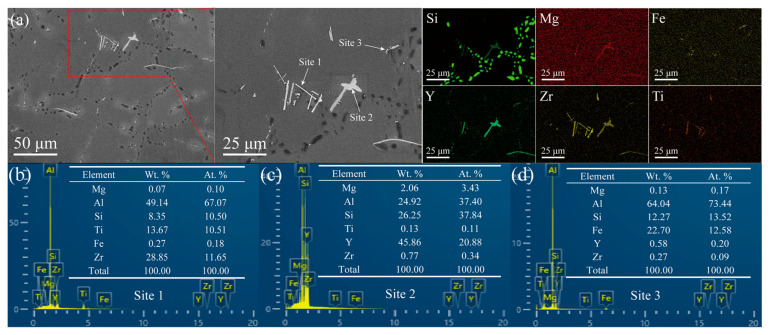
(**a**) SEM microstructures of AZ2Y3 alloy sample and (**b**–**d**) EDS spectra from site 1 to site 3 located in (**a**).

To gain a clear understanding of the distribution of Zr/Y within the alloy, the EDS energy spectrum is presented in Figure 3. The characteristics of the coarse primary dendrites in the primary α-Al phases of the A0 and AY3 alloys can be identified from the EDS elemental distribution mapping shown in Figure 3a,c. Similarly, the finer and equiaxed features of the α-Al phases in the alloy samples AZ2 and AZ2Y3 are also evident (Figure 3b,d). The Al–Si binary phase diagram indicates that Si’s solubility in Al is 1.65 wt% at the eutectic temperature of 577 °C, decreasing to 0.05 wt% at room temperature (≈25 °C). As shown in Figure 3, Si mainly forms a pure phase within the eutectic regions of the alloys. Furthermore, the addition of 0.2 wt% Zr and 0.3 wt% Y results in the formation of Zr-rich and Y-rich phases, respectively. Figure 4 presents the phase composition of the AZ2Y3 alloy as determined by EDS point analysis. As shown in Figure 4a, rod-like Zr–Ti phases (site 1) were observed and identified as (Al, Si)_3_(Zr, Ti) phases, consistent with previous studies on aluminum alloys. Additionally, irregular block-shaped Y-rich phases (site 2) were identified as Al_2_Si_2_Y phases. The fine needle-like structures (site 3) are likely to be identified as β-Al_5_FeSi.

### 3.2. Corrosion Behavior

#### 3.2.1. EIS Analysis

**Figure 5:**
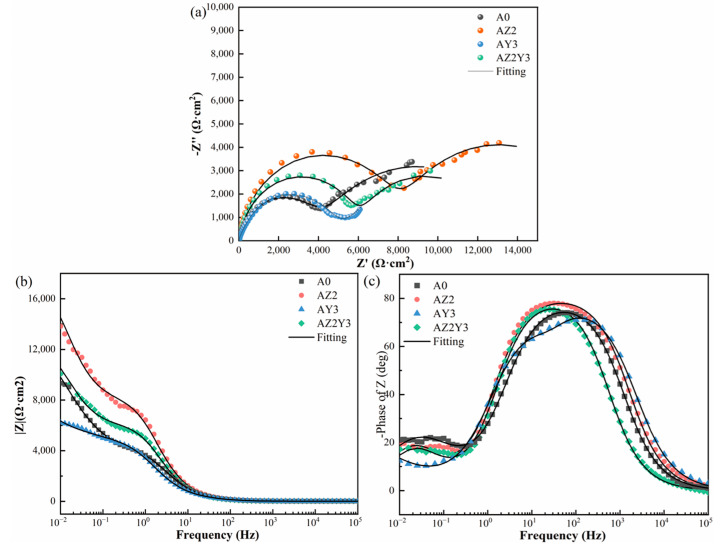
(**a**) Nyquist plots, (**b**) Bode impedance plots, and (**c**) Bode phase angle plots of four T6-treated Al–Si–Mg alloys.

Incorporating Zr and/or Y can substantially alter the morphology of the α-Al and Si phases, thereby affecting the corrosion properties of alloys. EIS experiments were performed at 25 °C with samples submerged in a 3.5% NaCl solution for 7 days to examine the effect of these additions on corrosion behavior. The experimental results, including Nyquist plots, Bode impedance plots, Bode phase angle plots, and their respective fittings, are presented in Figure 5. As shown in Figure 5a, there are two capacitor loops in the Nyquist plot, corresponding to two time constants in the Bode plot. In the high-frequency range, the capacitor loop reflects the electrochemical behavior of the passivation film, whereas in the low-frequency range, it is linked to the local corrosion process (Al—Al^3+^ process) [27,28]. The capacitive arc diameter indicates the corrosion resistance of alloys, where a larger diameter reflects better resistance [29]. Additionally, the diameters of the two capacitive loops for AZ2 are larger than those of the other samples. In contrast, the addition of 0.3 wt% Y results in no significant change in the capacitive loop diameter in the low-frequency (LF) region, but leads to a reduction in the capacitive loop diameter in the high-frequency (HF) region compared to the A0 alloy. The higher the impedance value (|Z|) in the LF region of the Bode plot, the better the corrosion resistance. Figure 5b shows the |Z|_f=0.01_ values for the alloys: A0 (9326 Ω cm^2^), AZ2 (13818 Ω cm^2^), AY3 (6225 Ω cm^2^), and AZ2Y3 (10,074 Ω cm^2^). This trend aligns with the changes observed in the diameter of the capacitive arc. The phase angle located in the intermediate frequency range (10–10^3^ Hz) indicates the capacitance behavior. A reduced phase angle height and width signify increased capacitance, which is associated with decreased charge transfer resistance and enhanced surface charge accumulation, thus expediting corrosion.

**Figure 6:**
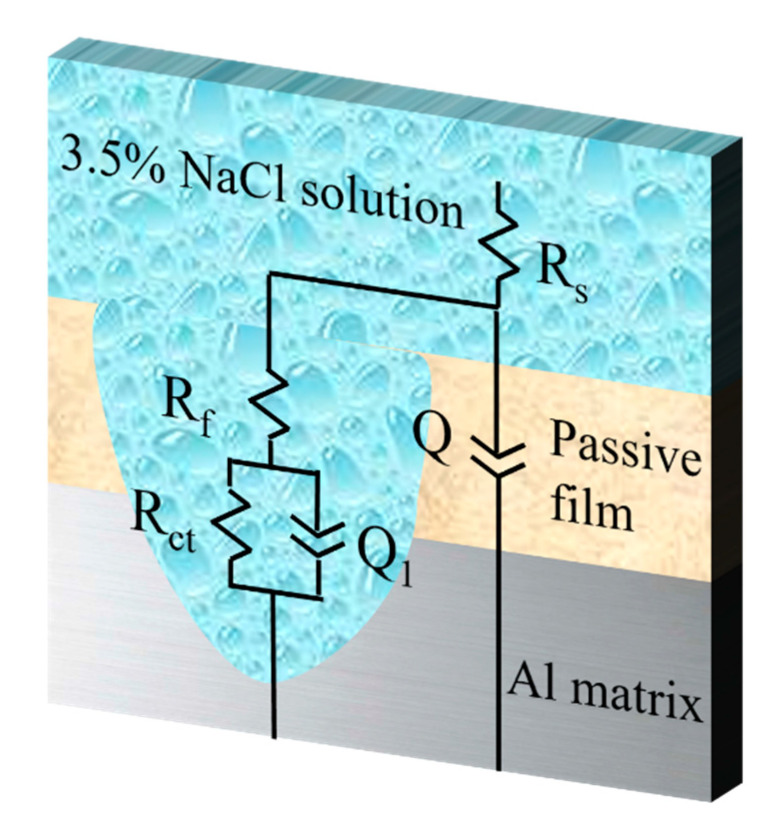
Equivalent electrical circuit used for fitting the electrochemical impedance data.

Based on the above analysis, the EIS results depicted in Figure 5 can be effectively modeled using the equivalent circuit diagram illustrated in Figure 6. The parameters derived from this fitting process are detailed in Table 3. R_s_ denotes the solution resistance, R_f_ represents the surface film resistance, and R_ct_ is the charge transfer resistance. Meanwhile, Q and Q_1_ correspond to the constant phase elements (CPEs) of the surface layer and the double layer, respectively [27]. The characteristics of the alloys on the surface layer can be indicated by the R_f_ and Q values. High R_f_ and low Q values suggest enhanced corrosion resistance of the surface layer. In addition, R_ct_ and Q_1_ offer valuable information regarding the electrode–electrolyte interface and the capacitance of the double layers. It is worth noting that the R_ct_ value has an inverse relationship with the corrosion rate. Consequently, higher R_ct_ values combined with lower Q_1_ values indicate that the alloy samples possess excellent corrosion resistance.

The R_f_ value of AZ2 alloy is the highest at 8272 Ω cm^2^, while the R_f_ value of alloy AY3 is the lowest at 4161 Ω cm^2^. Furthermore, the value of R_ct_ exhibits a trend similar to that of the R_f_ values. The capacitance of the passive film can be calculated by utilizing the curve-fitted Q in conjunction with Equations (3) and (4).

(3) $$C_{\mathrm{eff}}{=Q}^{1/n}{\left(R_{s}^{-1}{+R}_{f}^{-1}\right)}^{(n-1)/n}$$

(4) $$d=\varepsilon_{0}\varepsilon_{r}/C_{\mathrm{eff}}$$

C_eff_ denotes the effective capacitance of the passive film, and ε_0_ and ε_r_ correspond to the dielectric constant of a vacuum and the relative constant for the partially hydrated layer, respectively. d denotes the thickness of the passive film [29].

The passive film thicknesses for the alloys A0, AZ2, AY3, and AZ2Y3 are 1.13 nm, 1.58 nm, 0.74 nm, and 1.21 nm, respectively. The passive film’s thickness indirectly indicates its protective ability, with increased thickness providing better corrosion resistance. The inclusion of 0.2 wt% Zr significantly enhances the thickness of the passive film.

#### 3.2.2. Potentiodynamic Polarization Analysis

**Figure 7:**
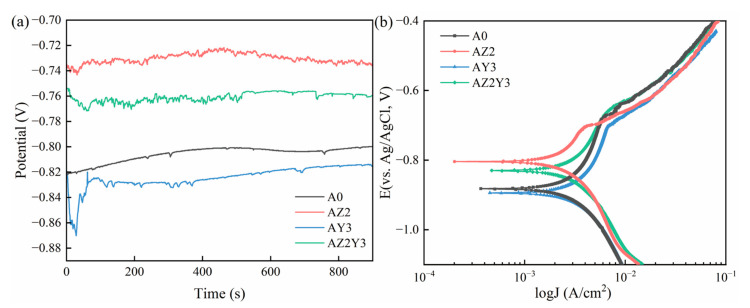
OCP and potentiodynamic polarization curves of four alloys after exposure to 3.5 wt% NaCl solution for 7 days: (**a**) OCP curve; (**b**) potentiodynamic polarization curve.

Figure 7a shows the OCP curve of four samples monitored in 3.5% NaCl solution for 900 s. It can be seen from Figure 7a that the E_ocp_ values of four alloys change at the initial stage of the immersion and reach a stable state after 900 s. The E_ocp_ value reflects the reactivity of the metal surface. The E_ocp_ values of the AZ2 and AZ2Y3 alloys were positively shifted compared to the A356 and AY3 alloys, indicating greater thermodynamic stability in a 3.5% NaCl solution.

Figure 7b shows the polarization curves of four samples after a 7-day immersion in a 3.5 wt% NaCl solution. To further corroborate the experimental findings, the Tafel extrapolation method was employed to fit the polarization curves (Figure 7b). The electrochemical parameters obtained from the potentiodynamic polarization curves are listed in Table 4. The anodic process of Al alloys in a sodium chloride medium is relatively complex, involving the formation of corrosion products. In contrast, the cathodic process is mainly the reduction of oxygen. The corresponding chemical reactions are as follows:

(5) $$\mathrm{Al}\to {\mathrm{Al}}^{3+}+3e^{-}$$

(6) $${\mathrm{Al}}^{3+}+3{\mathrm{OH}}^{-}\to \mathrm{Al}(\mathrm{OH}{)}_{3}$$

(7) $$2\mathrm{Al}(\mathrm{OH}{)}_{3}\to Al_{2}O_{3}+3H_{2}O$$

(8) $$O_{2}+2H_{2}O+4e^{-}\to 4{OH}^{-}$$

Therefore, corrosion potential values (E_corr_) and corrosion current density (J_corr_) are obtained using the Tafel cathodic extrapolation method. And the polarization resistance (R_p_) and the corrosion rate (P_i_) are derived from Equations (1) and (2) and the corresponding data. E_corr_ is commonly used as an indicator of corrosion tendency from a thermodynamic perspective, while J_corr_ serves as an indicator from a kinetic perspective [30]. A high R_p_ value and a low P_i_ value indicate better corrosion resistance of the alloys. For the A356 alloy, E_corr_ is −0.830 V and J_corr_ is 1.07 μA/cm^2^. With the addition of Zr, the E_corr_ shifts in a positive direction, with J_corr_ decreasing. Specifically, for the AZ2 alloy, E_corr_ is −0.779 V and J_corr_ is 0.51 μA/cm^2^, while for the AZ2Y3 alloy, E_corr_ is −0.789 V and J_corr_ is 0.71 μA/cm^2^. In contrast, the J_corr_ increased when 0.3 wt% Y was added alone, indicating a decline in corrosion resistance. For the AY3 alloy, E_corr_ was −0.896 V and J_corr_ was 2.01 μA/cm^2^. As demonstrated in Table 4, there is an inverse relationship between the R_p_ values and the J_corr_ values, suggesting that R_p_ can serve as an indicator of corrosion resistance trends for the tested alloys. The ranking of corrosion rates is AZ2 < AZ2Y3 < A0 < AY3. The above analysis shows that the AZ2 alloy has the highest corrosion resistance and the lowest corrosion rate.

#### 3.2.3. Immersion Corrosion Test

**Figure 8:**
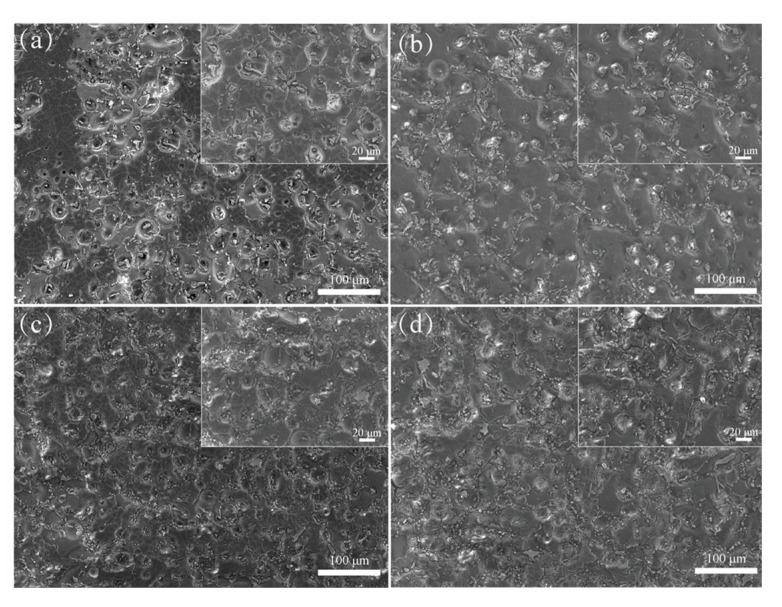
SEM micrographs illustrating corrosion microstructures after 28 days of immersion in 3.5 wt% NaCl solution: (**a**) A0, (**b**) AZ2, (**c**) AY3, and (**d**) AZ2Y3.

In order to verify the results of the electrochemical corrosion behavior study, an immersion corrosion test was carried out in 3.5 wt% NaCl solution for 28 days. Figure 8 shows the microscopic corrosion morphologies of the test alloys after the removal of corrosion products. In the A0 alloy (Figure 8a), the surface exhibits heterogeneous corrosion characterized by large and small corrosion pits distributed unevenly across the surface.

The α-Al in close proximity to the eutectic Si has been dissolved, while the eutectic Si within these regions remains unaffected. This observation suggests that the eutectic Si phase exhibits superior electrochemical stability compared to the α-Al phase, thereby facilitating galvanic interaction between the two phases. As a consequence, small corrosion pits start to form around the eutectic Si particles and progressively evolve into larger and deeper pits. In the AZ2 alloy (Figure 8b), no evident corrosion pits were observed, with slight intergranular corrosion observed at the grain boundaries. Consequently, the corroded surface exhibited a relatively smooth morphology. This indicates that the incorporation of 0.2 wt% Zr substantially reduces the degree of localized corrosion in the AZ2 alloy. In contrast, the AY3 alloy (Figure 8c) exhibits significant intergranular corrosion cracks in the α-Al matrix encircling the eutectic Si particles. The corrosion is uniformly distributed without large or deep pits, unlike the A0 alloy. Figure 8d illustrates that the simultaneous addition of Zr and Y results in corrosion pit sizes similar to those observed in the AZ2 alloy. However, the α-Al matrix surrounding the eutectic Si particles exhibits more pronounced corrosion compared to the AZ2 alloy.

**Figure 9:**
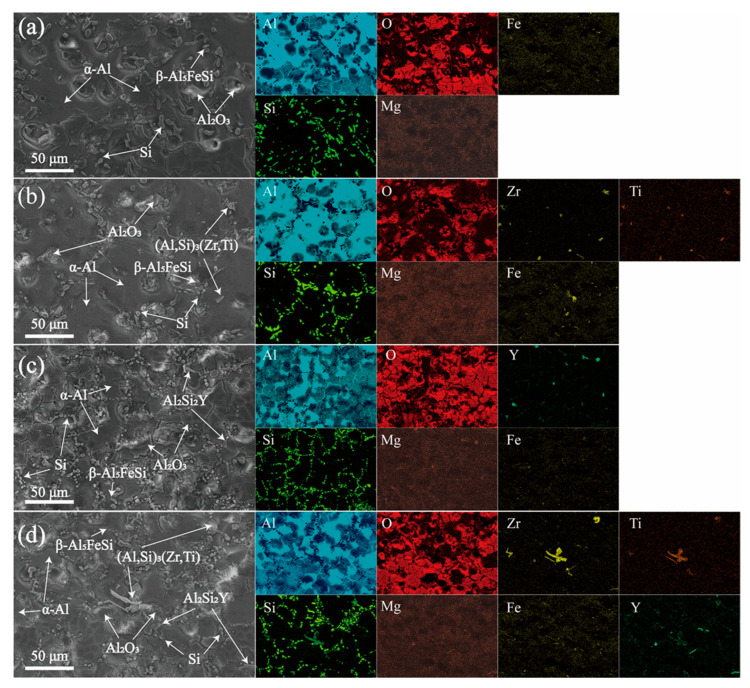
SEM micrographs of alloys after immersion in 3.5 wt% NaCl solution for 28 days: (**a**) A0, (**b**) AZ2, (**c**) AY3, and (**d**) AZ2Y3.

Figure 9 shows the SEM micrographs of four test alloys after 28 days of immersion in 3.5 wt% NaCl solution. In the A0 alloy (Figure 9a), pitting and intergranular corrosion are the predominant forms of corrosion. Furthermore, EDS analysis indicates that the aluminum–oxygen oxide layer in the Al–Si eutectic region is prone to flaking during corrosion. In the AZ2 alloy (Figure 9b), the intergranular and intragranular (Al, Si)_3_(Zr, Ti) precipitates refine the α-Al structure, thereby enhancing corrosion resistance. Minor pitting and intergranular corrosion are observed under these conditions. The incorporation of 0.3 wt% Y modifies the plate-like Si into a fibrous-like morphology, which becomes further refined after T6 treatment (Figure 9c,d). This results in an expanded network of intergranular corrosion channels, promoting crack propagation perpendicular to the eutectic region and consequently leading to diminished corrosion protection.

### 3.3. XPS Analysis

**Figure 10:**
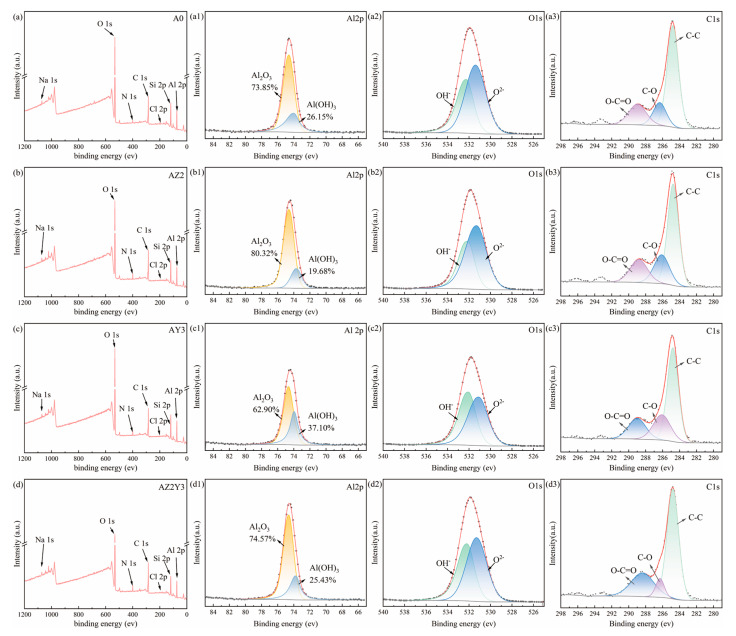
XPS survey of four T6-treated samples after being immersed in 3.5 wt% NaCl solution for 7 days: (**a**–**a3**) A0 alloy; (**b**–**b3**) AZ2 alloy; (**c**–**c3**) AY3 alloy, and (**d**–**d3**) AZ2Y3 alloy.

XPS analysis was employed to further confirm the chemical composition of the corrosion products of four alloys, as illustrated in Figure 10. After 7 days of immersion in a 3.5% NaCl solution, the surfaces of the samples were found to contain elements such as C, O, N, Na, Cl, Al, and Si. The elements C and N are attributed to the dissolution of CO_2_ and N_2_ from the air into the 3.5% NaCl solution. The elements Na, Cl, and Al originate from the corrosion products. Moreover, due to the substantial amount of eutectic Si in the A0 alloy, the Si element was also observed. The Al 2p spectra (Figure 10(a1–d1)) consist of two characteristic peaks whose binding energies are 74.6 eV and 73.7 eV [4], and the corresponding products are Al_2_O_3_ and Al(OH)_3_, respectively. Similarly, the O 1s spectra (Figure 10(a2–d2)) of these materials are composed of two characteristic peaks, OH^-^ with a binding energy of 532.6 eV and O^2-^ with a binding energy of 531.1 eV [31]. As shown in Figure 10(a3–d3), C exists in three forms (Figure 10(a3–d3)), C-C, C-O and O-C=O, with the highest concentration being C-C. The content of aluminum oxide plays a critical role in enhancing the corrosion resistance of aluminum alloys. The estimated aluminum oxide contents in the A0, AZ2, AY3, and AZ2Y3 alloys are 73.85%, 80.32%, 62.90%, and 74.57%, respectively.

XPS analysis indicates that Al_2_O_3_ is the primary corrosion product, with Zr and Y additions enhancing its formation. Moreover, the content of Al_2_O_3_ is the highest in the AZ2 alloy. The α-Al matrix experiences selective dissolution when the alloy is in direct contact with the electrolyte. Severe localized corrosion was observed at the interface between the unrefined α-Al and eutectic Si particles within the T6-treated A0 alloy. Under passivating conditions (3.5 wt% NaCl solution), the formation of the passive film is promoted due to the refined α-Al grains, thereby enhancing its protective capacity and reducing corrosion within the α-Al matrix.

**Figure 11:**
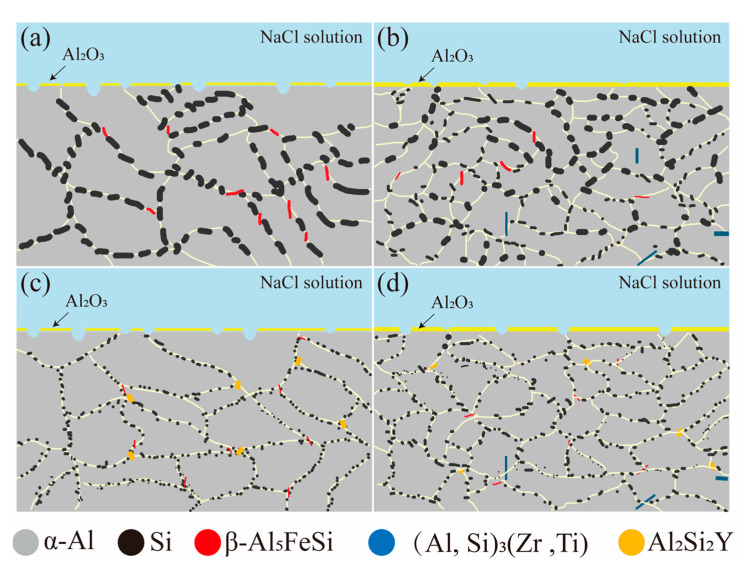
Schematic illustrations showing the corrosion mechanism of A0 series alloys in 3.5 wt% NaCl solution: (**a**) A0; (**b**) AZ2; (**c**) AY3; (**d**) AZ2Y3.

Figure 11 is a schematic diagram illustrating the corrosion mechanism. The alloy’s corrosion resistance is primarily determined by its microstructure. Previous studies [23] have extensively examined the mechanisms by which Zr refines α-Al grains and Y modifies eutectic Si phases. In the A0 alloy, the Si and β-Al_5_FeSi phases exhibit significantly higher surface potentials compared to the α-Al phases, causing them to act as cathodes that attract electrons and initiate corrosion reactions with the α-Al phases [19]. Corrosion proceeds as the anodic α-Al phases gradually dissolve. Upon reaching a certain extent of corrosion, the exposed cathodic phases are eventually removed from the corrosion pits. The addition of 0.2 wt% Zr refined the α-Al grains, allowing the analysis of grain size impact on corrosion rate through Equation (9) [7].

(9) $$i_{corr}=a+bd^{-\frac{1}{2}}$$

Constants a and b are associated with materials and corrosive environments. The variable ‘d’ denotes the grain boundary length.

The distribution of the passive film on the alloy surface becomes more uniform after the refinement treatment. This is because the refined α-Al grains facilitate the formation of a passivation film layer (Figure 11b). As a result, the protective effect of this passivation film on the α-Al matrix is enhanced, leading to a significant reduction in the corrosion of the α-Al matrix.

As previously discussed, the addition of 0.3 wt% Y can modify the eutectic Si significantly, indicating that a higher distribution density of eutectic Si particles appears in the presence of Y. Consequently, more galvanic couplings occur in the AY3 and AZ2Y3 alloys relative to the A0 and AZ2 alloys (Figure 11c,d). The eutectic Si phase, being more noble than the surrounding eutectic α-Al phase, acts as a local cathode, leading to increased galvanic activity [21]. This explains the higher J_corr_ values observed with the addition of Y.

Furthermore, the negative electrochemical potential of Y (−2.379 V) is comparable to that of Mg (−2.372 V) but significantly lower than those of Al (−1.662 V), Si (−1.24 V), and Zr (−1.553 V) [32]. Additionally, while there is no specific data on the electrochemical potential of Al–Si–Y intermetallics, it can be inferred that these compounds may have potentials similar to their constituent elements. Therefore, the Al_2_Si_2_Y intermetallic phases function as anodes, sacrificing themselves to protect the α-Al matrix from corrosion. This phenomenon also accounts for the observed differences in the size of the Al_2_Si_2_Y phase in Figure 3c,d and Figure 9c,d.

## 4. Conclusions

In this study, the microstructure evolution and corrosion behavior of T6-treated Al–Si–Mg alloys were investigated in the presence of Zr and Y additions. The analysis was conducted using a combination of XRD, OM, SEM, XPS, and electrochemical experiments. The addition of 0.2 wt.% Zr into the Al–Si–Mg alloy significantly refined the size of α-Al dendrites. Furthermore, the incorporation of Y facilitates the transformation of rod-like eutectic Si particles into a more spherical morphology, reducing the major axis of these particles from 6.18 ± 4.38 μm to 2.47 ± 1.09 μm. The corrosion primarily occurs in the eutectic regions of the alloys during immersion, where the eutectic α-Al phases predominantly dissolve while the eutectic Si phases remain intact. This observation confirms that the Si phase exhibits higher nobility compared to the α-Al phase, leading to the formation of galvanic couplings between the eutectic Si and α-Al phases. Both the electrochemical testing results and XPS show that the introduction of Zr into Al–Si–Mg alloy promotes the formation of a thicker and more uniform passive film. Also, the addition of 0.3 wt.% Y to the Al–Si–Mg alloy results in a higher density of eutectic Si particles, leading to an augmented network of intergranular corrosion channels and more galvanic couplings, thereby reducing the corrosion resistance.

## Figures and Tables

**Table 1:** Chemical composition (wt%) detected by ICP of four alloys. Reprinted from Ref. [23].

Alloy	Si	Mg	Fe	Ti	Y	Zr	Al
A0	7.03	0.29	0.095	0.13	0	0	Balance
AZ2	6.82	0.28	0.14	0.10	0	0.2	Balance
AY3	6.81	0.31	0.094	0.13	0.29	0	Balance
AZ2Y3	6.86	0.29	0.14	0.11	0.27	0.19	Balance

**Table 2:** Statistical analysis of eutectic Si particles for four samples in Figure 2.

Alloy	Major Axis (μm)	Minor Axis (μm)	Aspect Ratio
A0	6.18 ± 4.38	2.59 ± 1.02	2.45 ± 1.61
AZ2	5.35 ± 3.37	2.24 ± 0.81	2.46 ± 1.48
AY3	2.47 ± 1.09	1.76 ± 0.69	1.41 ± 0.36
AZ2Y3	3.17 ± 1.59	2.08 ± 0.80	1.52 ± 0.49

**Table 3:** The EIS fitting parameters obtained from Figure 5.

Alloy	R_s_ (Ω cm^2^)	Q (μS s^n^ cm^−2^)	n	R_f_ (Ω cm^2^)	Q_1_ (μS s^n^ cm^−2^)	n_1_	R_ct_ (Ω cm^2^)
A0	9.92	22.0	0.90	4269	990	0.71	8460
AZ2	9.77	17.5	0.91	8272	645	0.82	10240
AY3	9.83	34.2	0.90	4161	1130	0.80	6820
AZ2Y3	10.11	20.1	0.92	6122	795	0.83	9956

**Table 4:** Corrosion characteristics obtained from potentiodynamic polarization measurement in Figure 7b.

Alloy	E_corr_ (V)	J_corr_ (μA/cm^2^)	R_p_ (KΩ cm^2^)	P_i_ (mm/year)
A0	−0.830	1.07	27.5	0.0244
AZ2	−0.779	0.51	72.5	0.0117
AY3	−0.896	2.01	22.4	0.0459
AZ2Y3	−0.789	0.71	40.3	0.0162

## Data Availability

The data presented in this study are available on request from the corresponding author due to the ongoing nature of the study, which precludes disclosure at present.

## References

[1] Pan S., Liu Y., Yang B., Fu Y., Gao M., Guan R. (2023). Effect of Stirring Speed and Temperature on the Microstructure and Mechanical Properties of A356 Alloy under Vacuum. Vacuum.

[2] Teng D., Zhang G., Zhang S., Li J., Jia H., He Q., Guan R. (2024). Microstructure Evolution and Strengthening Mechanism of A356/Al-X-Ce(Ti, C) System by Inoculation Treatment. J. Mater. Res. Technol..

[3] Kang H., Jang H., Oh S., Yoon P., Lee G., Park J., Kim E., Choi Y. (2021). Effects of Solution Treatment Temperature and Time on the Porosities and Mechanical Properties of Vacuum Die-Casted and T6 Heat-Treated Al–Si–Mg Alloy. Vacuum.

[4] Xu S.-L., Jia H.-L., Zha M., Zhou X.-L., Gao D., Ma P.-K., Wang D. (2024). The Effect of B and Sb on the Corrosion Behavior of T6-Treated Al–Si–Mg Alloys. J. Mater. Res. Technol..

[5] Zou Y., Liu Q., Jia Z., Xing Y., Ding L., Wang X. (2017). The Intergranular Corrosion Behavior of 6000-Series Alloys with Different Mg/Si and Cu Content. Appl. Surf. Sci..

[6] Gollapudi S. (2012). Grain Size Distribution Effects on the Corrosion Behaviour of Materials. Corros. Sci..

[7] Ralston K.D., Birbilis N., Davies C.H.J. (2010). Revealing the Relationship between Grain Size and Corrosion Rate of Metals. Scr. Mater..

[8] Chen Z., Kang H., Fan G., Li J., Lu Y., Jie J., Zhang Y., Li T., Jian X., Wang T. (2016). Grain Refinement of Hypoeutectic Al-Si Alloys with B. ACTA Mater..

[9] Timpel M., Wanderka N., Schlesiger R., Yamamoto T., Lazarev N., Isheim D., Schmitz G., Matsumura S., Banhart J. (2012). The Role of Strontium in Modifying Aluminium–Silicon Alloys. ACTA Mater..

[10] Zheng Q., Zhang L., Jiang H., Zhao J., He J. (2020). Effect Mechanisms of Micro-Alloying Element La on Microstructure and Mechanical Properties of Hypoeutectic Al-Si Alloys. J. Mater. Sci. Technol..

[11] Kang J., Su R., Wu D.Y., Liu C.H., Li T., Wang L.S., Narayanaswamy B. (2019). Synergistic Effects of Ce and Mg on the Microstructure and Tensile Properties of Al-7Si-0.3Mg-0.2Fe Alloy. J. Alloys Compd..

[12] Mao F., Yan G., Xuan Z., Cao Z., Wang T. (2015). Effect of Eu Addition on the Microstructures and Mechanical Properties of A356 Aluminum Alloys. J. Alloys Compd..

[13] Limmaneevichitr C., Eidhed W. (2003). Fading Mechanism of Grain Refinement of Aluminum–Silicon Alloy with Al–Ti–B Grain Refiners. Mater. Sci. Eng. A.

[14] Liao H., Sun G. (2003). Mutual Poisoning Effect between Sr and B in Al–Si Casting Alloys. Scr. Mater..

[15] Li B., Wang H., Jie J., Wei Z. (2011). Effects of Yttrium and Heat Treatment on the Microstructure and Tensile Properties of Al–7.5Si–0.5Mg Alloy. Mater. Des..

[16] Liu Z., Hu Y. (2008). Effect of Yttrium on the Microstructure of a Semi-Solid A356 Al Alloy. Rare Met..

[17] Colombo M., Gariboldi E., Morri A., Tonelli D. (2019). SKPFM Investigations of Intermetallic Compounds of Innovative Er- and Zr-Containing Al–Si–Mg Alloys and Their Influence on Corrosion Localization in Saline Solution. Mater. Corros..

[18] Zou Y., Yan H., Yu B., Hu Z. (2019). Effect of Rare Earth Yb on Microstructure and Corrosion Resistance of ADC12 Aluminum Alloy. Intermetallics.

[19] Farahany S., Ourdjini A., Bakhsheshi-rad H.R. (2016). Microstructure, Mechanical Properties and Corrosion Behavior of Al–Si–Cu–Zn–X (X = Bi, Sb, Sr) Die Cast Alloy. Trans. Nonferrous Met. Soc. China.

[20] Ru Z., Wei L., Liu M., Pi Z., Han C., Qiu M., Li E., Huang H., Li D. (2025). Effect of Y and Zr addition on the intergranular corrosion of Al-Mg alloy after sensitization treatment. Micron.

[21] Ma Y., Wang M., Liu Y., Cai B. (2022). Microstructures and Corrosion Behaviors of Al–6.5Si–0.45Mg–*x*Sc Casting Alloy. Trans. Nonferrous Met. Soc. China.

[22] Ma Y., Liu Y., Wang M. (2022). Microstructures and Corrosion Resistances of Hypoeutectic Al-6.5Si-0.45 Mg Casting Alloy with Addition of Sc and Zr. Mater. Chem. Phys..

[23] Ye P., Jiang F., Wu F., Ye K., Fan Y. (2024). Effects of Zr and Y Additions on Microstructure and Mechanical Properties of Cast A356 Alloy. J. Mater. Res. Technol..

[24] Liu M., Schmutz P., Uggowitzer P.J., Song G., Atrens A. (2010). The Influence of Yttrium (Y) on the Corrosion of Mg–Y Binary Alloys. Corros. Sci..

[25] Zhao M.-C., Schmutz P., Brunner S., Liu M., Song G., Atrens A. (2009). An Exploratory Study of the Corrosion of Mg Alloys during Interrupted Salt Spray Testing. Corros. Sci..

[26] Fang X., Zhang T., Dong B., Yuan Z., Huang Z., Yan F., Zu F. (2024). Simultaneous Refinement of α-Al and Modification of Si in Al–Si Alloy Achieved via the Addition of Y and Zr. J. Mater. Res. Technol..

[27] Zheng Q., Wu J., Jiang H., Zhang L., Zhao J., He J. (2021). Effect of Micro-Alloying Element La on Corrosion Behavior of Al-Mg-Si Alloys. Corros. Sci..

[28] Zhang B., Wang J., Wu B., Guo X.W., Wang Y.J., Chen D., Zhang Y.C., Du K., Oguzie E.E., Ma X.L. (2018). Unmasking Chloride Attack on the Passive Film of Metals. Nat. Commun..

[29] Jiang Y., Dai S., Chen Q., Xin X., Song Z., Chen Z., Wu C., Ren X., Meng C. (2025). Innovative Heterostructure Enhance Mechanical Strength and Corrosion Resistance in A356/6061 Dissimilar Aluminum Alloy MIG Welded Joints. Vacuum.

[30] Hirschorn B., Orazem M.E., Tribollet B., Vivier V., Frateur I., Musiani M. (2010). Determination of Effective Capacitance and Film Thickness from Constant-Phase-Element Parameters. Electrochim. Acta.

[31] Geng R., Jia S.-Q., Qiu F., Zhao Q.-L., Jiang Q.-C. (2020). Effects of Nanosized TiC and TiB2 Particles on the Corrosion Behavior of Al-Mg-Si Alloy. Corros. Sci..

[32] Milazzo G., Caroli S., Braun R.D. (1978). Tables of Standard Electrode Potentials. J. Electrochem. Soc..

